# Contributions of prognostic factors for poor outcome in primary care low back pain patients

**DOI:** 10.1016/j.ejpain.2010.07.008

**Published:** 2011-03

**Authors:** Kate M. Dunn, Kelvin P. Jordan, Peter R. Croft

**Affiliations:** Arthritis Research UK Primary Care Centre, Keele University, UK

**Keywords:** Back pain, Epidemiology, Cohort study, Primary health care, Targeting

## Abstract

**Background:**

Back pain is common and some sufferers consult GPs, yet many sufferers develop persistent problems. Combining information on risk of persistence and prognostic indicator prevalence provides more information on potential intervention targets than risk estimates alone.

**Aims:**

To determine the proportion of primary care back pain patients with persistent problems whose outcome is related to measurable prognostic factors.

**Methods:**

Prospective cohort study of back pain patients (30–59 years) at five general practices in Staffordshire, UK (*n* = 389). Baseline factors (demographic; episode duration; symptom severity; pain widespreadness; anxiety; depression; catastrophising; fear-avoidance; self-rated health) were assessed for their association with disabling and limiting pain after 12-months. The proportion of those with persistent problems whose outcome was related to each factor was calculated.

**Results:**

Prevalence of prognostic factors ranged from 23% to 87%. Strongest predictors were unemployment (adjusted relative risk (RR) 4.2; 95% CI 2.0, 8.5) and high pain intensity (4.1; 1.7, 9.9). The largest proportions of persistent problems were related to high pain intensity (68%; 95% CI 27, 87%) and unemployment (64%; 33, 82%). Combining these indicated that 85% of poor back pain outcome is related to these two factors. Poor self-rated health, functional disability, upper body pain and pain bothersomeness were related with outcome for over 40% of those with persistent problems.

**Conclusions:**

Several factors increased risk of poor outcome in back pain patients, notably high pain and unemployment. These risks in combination with high prevalence of risk factors in this population distinguish factors that can help identify targets or sub-groups for intervention.

## 1 Introduction

Back pain is common in the general population; around 30% have low back pain (LBP) during any 1 month (Papageorgiou et al., 1995, Webb et al., 2003), and at least 60% of adults experience LBP during their lifetime (Papageorgiou et al., 1995, Hillman et al., 1996, Walsh et al., 1992). LBP has important consequences for sufferers. It is linked to the onset of psychological problems such as depression (Carroll et al., 2003) and is also a major cause of work absence, leading to substantial economic consequences (Wynne-Jones et al., 2008). LBP is therefore a significant public health problem.

Although many LBP sufferers do not recover completely (Hestbaek et al., 2003), fewer than one-third seek healthcare (Carey et al., 1996, McKinnon et al., 1997). As LBP is so common, this means 6–9% of adults seek healthcare for LBP annually (Croft et al., 1998, Dunn and Croft, 2005, Royal College of General Practitioners, 1995). It is therefore a considerable burden on primary care, where most LBP management occurs, and several studies have investigated prognosis in primary care (Lanier and Stockton, 1988, Von Korff et al., 1993, Coste et al., 1994, Klenerman et al., 1995, Croft et al., 1998, van den Hoogen et al., 1998, Reis et al., 1999, Schiøttz-Christensen et al., 1999, Carey et al., 2000, Nyiendo et al., 2001, Burton et al., 2004, Jones et al., 2006, Mallen et al., 2007). These studies have focused on prognostic ability, including factors measuring pain intensity and widespreadness, disability and psychological status, but have not investigated the proportion of poor prognosis that is related to each factor.

Population attributable fractions (PAFs) are used in aetiological research to estimate the public health impact of removing a putative cause of disease from a population. They depend on the strength of association between cause and effect, and on the population prevalence of the causal factor – because smoking is common, the proportion of lung cancer attributed to it is high and the effect of removing smoking on lung cancer occurrence is substantial. This is an advantage over presentation of relative risks (RRs), as rare exposures with high RRs may not present good population intervention targets. Such calculations can also be applied to prognostic factors in presenting illness or established disease – the population in this case is everyone with the illness, and the calculation refers to outcome rather than disease onset. When identifying sub-groups for treatment targeting, factors identifying high-risk patients are not necessarily causal, and therefore standard PAF interpretation – that the relationship being quantified is causal – might not apply. However, the PAF calculation itself provides useful information on prognostic markers, or groups in which to target interventions, and gives clear methods for comparing the impact of new interventions. For example, if two prognostic indicators have similar associations with outcome, but one is common and the other rare, intervening on the common factor would have greater public health impact. We therefore aimed to determine the risk factors for poor prognosis – and their relative contributions to outcome – in adults consulting with LBP in primary care.

## 2 Methods

We included patients from the Backpain Research in North Staffordshire (BaRNS) Study, a prospective cohort of primary care LBP patients (Dunn and Croft, 2005). The North Staffordshire Local Research Ethics Committee approved this study.

### 2.1 Patients and setting

**Fig. 1:**
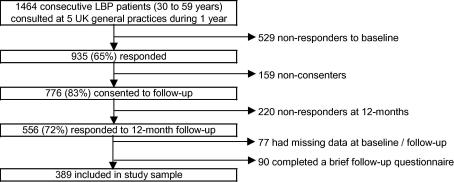
Flow chart showing inclusion in study sample.

Participants were recruited from five computerised General Practices in North Staffordshire, UK, covering a socio-economically and geographically heterogeneous population (Noble et al., 2004). Consecutive patients aged 30–59 years consulting their General Practitioner (GP) with LBP during the 12-months following October 2001 were sent a self-completion questionnaire. Patients were identified through the use of morbidity codes indicating a LBP consultation at the general practice. Further details of patient recruitment are reported elsewhere (Dunn and Croft, 2005). Patients returning the baseline questionnaire (65%, *n* = 935) and consenting to further contact (83%, *n* = 776) were sent a 12-month follow-up questionnaire. Information was available on 72% at 12-months, of whom 389 provided full information (see Fig. 1). Included participants had similar baseline characteristics to the total baseline sample; their mean age (*n* = 389) was 46.7 years, compared with 45.6 for baseline responders (*n* = 935), 54.2% were female vs. 56.6%, mean pain intensity was 4.6 in both samples, mean modified Roland-Morris Disability (RMDQ) score was 10.0 vs. 9.7, and mean Hospital Anxiety and Depression (HADS) Scores were 8.6 (anxiety) and 7.2 (depression) in this sample vs. 8.6 and 7.1 in the total baseline sample. Included participants were also similar at follow-up to the group returning only the brief 12-month questionnaire (*n* = 90), with 26% of the brief responders saying that their back pain was very or extremely bothersome at 12-months, compared to 20% of the included sample.

### 2.2 Potential prognostic factors

The baseline questionnaire contained demographic items plus questions relating to LBP intensity, disability and psychological status. The reliability of these instruments has been established in a similar sample (Dunn et al., 2003). Age was dichotomised at the mid-point of the study sample, with older age being 45–59 years. Participants were asked for their highest educational qualification, and were categorised into those with and without education beyond age 16 years. People in employment who said that they were slightly or severely dissatisfied with their job were defined as being dissatisfied. Similarly, people who were not in employment who said that they were slightly or severely dissatisfied with not being employed were defined as being dissatisfied. These two variables were combined to produce a variable called satisfaction with work status. The definition of work absence due to LBP comprised people who were employed but currently off work due to low back pain plus people who were unemployed and reported that this was due to LBP.

LBP episode duration was measured using recall of time since the patients’ last pain-free month; long duration was defined as three or more years since the start of the episode (Dunn and Croft, 2006). Pain intensity was measured using the mean of three 0–10 numerical rating scales for least and usual LBP over the previous 2 weeks, and current LBP intensity; scores of five or more were defined as high pain intensity (Dunn et al., 2010). Functional disability was measured using the modified 23-item RMDQ (Patrick et al., 1995) with high functional disability defined as a score greater than 14 (Cherkin et al., 1998). Bothersome LBP was defined if people rated their pain during the previous 2 weeks as very much or extremely bothersome (Dunn and Croft, 2005). Information on previous LBP, and presence or absence of leg pain, distal leg pain and upper body pain (shoulder, arm, neck or head) over the previous 2 weeks was also collected.

Probable cases of clinical anxiety or depression were defined as scores of eleven or more on the HADS (Zigmond and Snaith, 1983). People were classified as catastrophisers if they felt that the pain was terrible and was never going to get any better based on a modified item from the Coping Strategies Questionnaire (Rosenstiel and Keefe, 1983). The use of single items to measure this construct has since been validated (Jensen et al., 2003), and the construct validity of this particular question has been established (Hill et al., 2008). Fear-avoidance beliefs were recorded if people stated that they could not do all the things normal people do because it is too easy for them to get injured, an item modified from the Tampa Scale for Kinesiophobia (Kori et al., 1990) and recommended for use as a single item (Vlaeyen et al., 2001). Self-reported health status was measured as reporting fair or poor on the general health perceptions question, and vitality was measured using with the vitality sub-scale, from the Short Form-36 questionnaire (Ware, 2000). For vitality, people below the bottom tertile (with scores less than 25) were defined as having low vitality.

### 2.3 Outcome

Outcome 12-months after baseline was measured using the Chronic Pain Grade (CPG; Von Korff et al., 1992). This classifies individuals into grades of chronic LBP: 0 (pain free), I (low disability, low intensity), II (low disability, high intensity), III (high disability, moderately limiting) and IV (high disability, severely limiting). A poor outcome is defined here as CPG IV (highly disabling and severely limiting LBP). This measure was chosen as the outcome as it was not included as a prognostic indicator in the current analysis.

### 2.4 Statistical analysis

Participants who returned the complete baseline and 12-month questionnaires were included in this analysis. Crude RRs with 95% confidence intervals (CI) were calculated for the associations between all potential prognostic indicators at baseline and 12-month outcome. Indicators that had a statistically significant association with outcome were then adjusted for potential confounders using Cox regression models with a constant time variable (Thompson et al., 1998). Six domains considered a priori to provide potential for confounding were adjusted for: episode duration, symptom severity (the strongest crude RR between pain intensity or disability), widespreadness of pain (the strongest of leg pain, distal leg pain or upper body pain), pain affect (the strongest crude RR between anxiety or depression), pain cognitions (the strongest crude RR between fear-avoidance or catastrophising) and self-reported health status. If none of the indicators within a particular confounding domain were statistically significantly associated with outcome in crude analyses, they were not considered to be confounders for the particular associations being investigated, and were not adjusted for. Indicators were not adjusted for other factors within the same domain. Additional inclusion of indicators within the same domain may have led to adjustment for factors lying on the same causal pathway, i.e. not confounders.

The proportion of those with persistent problems whose outcome was related to each factor was calculated using a PAF formula. Unadjusted figures were calculated using unadjusted RRs with the formula *p_r_*(RR − 1)/(*p_r_*(RR − 1)+1), where *p_r_* is the proportion of the population exposed (the proportion with the prognostic indicator). This formula is inappropriate when confounding exists and adjusted RRs are used as it can lead to biased estimates (Rockhill et al., 1998) and many prognostic indicators for LBP are likely to be inter-related. Therefore adjusted figures were calculated from the adjusted RRs using the more appropriate formula when confounding is likely to exist: *p_d_*((RR − 1)/RR), where *p_d_* is the proportion of those with a poor outcome at 12 months who were exposed. Ninety-five percent CIs were calculated using a method based on the Bonferroni inequality (Natarajan et al., 2007).

For the domains covering more than one risk factor, adjusted cumulative proportions based on combining the two strongest risk factors within each domain were calculated to ascertain the cumulative figure from each domain (Rockhill et al., 1998, Bruzzi et al., 1985). This was calculated using the formula $\sum_{i=0}^{k}p_{\mathrm{di}}\frac{\left({\mathrm{RR}}_{i}-1\right)}{{\mathrm{RR}}_{i}}$, where *p_di_* is the proportion of those with a poor outcome at 12 months in the *i*th exposure level across the two risk factors and RR_i_ is the adjusted RR for the *i*th exposure level compared to the group without either risk factor. This formula is recommended as being most valid when adjusted RRs are necessary due to confounding (Rockhill et al., 1998). These domain-specific proportions were adjusted for each of the other domains as before. A final adjusted cumulative proportion based on the two risk factors with the highest adjusted proportion (regardless of domain) was also calculated.

Analysis was carried out using Stata 9.0.

## 3 Results

The proportion of the 389 participants with each potential prognostic indicator at baseline is shown in Table 1. The most common factor was previous history of LBP (87%). Older age, leg pain and upper body pain were also present in over 60% of the sample. Two fifths of the sample reported having three or more years since the start of their back pain; of these, 40% reported having their pain for over 10 years. Among people with less than 3 years of pain, a third (33.5%) reported that their pain had started in the previous 3 months. All baseline prognostic indicators were present in over a fifth of the sample. At 12-months, 6.7% were pain free (CPG 0), 60.9% were in CPG I–II, 14.7% in CPG III and 17.7% of the sample had a poor outcome (CPG IV).

**Table 1:** Baseline characteristics of study sample (*n* = 389).

	No.	%
Gender (female)	211	54.2
Age group (older)	235	60.4
Education (left <16 years)	199	51.2
Not being in employment	140	36.0
Dissatisfaction with work status	164	42.2
Work absence	98	25.2
Previous history	338	86.9
Long duration	165	42.4
High functional disability	118	30.3
High pain intensity	181	46.5
Leg pain	258	66.3
Distal leg pain	165	42.4
Upper body pain	286	73.5
Bothersomeness	204	52.4
Anxiety	136	35.0
Depression	96	24.7
Fear-avoidance	90	23.1
Catastrophising	107	27.5
Poor self-rated health	152	39.1
Low vitality	107	27.5

### 3.1 Prognostic indicators

Table 2 presents the associations between potential baseline prognostic indicators and 12-month outcome. In unadjusted analyses, 17 baseline factors were significantly associated with highly disabling and severely limiting pain at follow-up. Not being in employment, work absence, high pain intensity or functional disability, bothersomeness and poor self-rated health indicated the strongest risk of a poor prognosis, all had statistically significant crude RRs above five. After adjustment for potential confounders, statistically significant associations remained for seven baseline factors: not being in employment, work absence, long episode duration, high functional disability, high pain intensity, anxiety and poor self-rated health. The strongest associations with outcome were found for not being in employment (RR 4.2; 95% CI 2.0, 8.5) and high pain intensity (RR 4.1; 95% CI 1.7, 9.9).

**Table 2:** Relative risks (RR) for the association between baseline risk factors and outcome at 12-months (*n* = 389).

Risk factor	Unadjusted		Adjusted	
	RR	(95% CI)	RR	(95% CI)

Gender (F)	1.03	(0.64, 1.66)	–	–
Age group (older)	1.02	(0.63, 1.65)	–	–
Less education	2.04	(1.23, 3.38)	1.20	(0.71, 2.04)
Not being in employment	9.38	(4.92, 17.87)	4.15	(2.03, 8.51)
Dissatisfaction with work status	3.89	(2.27, 6.65)	1.77	(0.99, 3.17)
Work absence	5.57	(3.39, 9.14)	2.40	(1.40, 4.10)
Previous history	1.01	(0.50, 2.03)	–	–
Long duration	2.39	(1.46, 3.90)	1.66	(1.01, 2.74)
High functional disability	6.51	(3.80, 11.14)	2.81	(1.42, 5.57)
High pain intensity	10.18	(4.66, 22.24)	4.13	(1.73, 9.88)
Leg pain	3.00	(1.53, 5.86)	1.34	(0.67, 2.68)
Distal leg pain	1.77	(1.10, 2.84)	0.99	(0.61, 1.62)
Upper body pain	3.78	(1.64, 8.74)	2.17	(0.93, 5.05)
Bothersomeness	6.05	(3.00, 12.18)	2.11	(0.92, 4.82)
Anxiety	4.56	(2.71, 7.67)	1.84	(1.05, 3.25)
Depression	3.97	(2.47, 6.39)	1.53	(0.90, 2.61)
Fear-avoidance	2.14	(1.32, 3.46)	1.08	(0.66, 1.78)
Catastrophising	4.94	(3.01, 8.11)	1.46	(0.83, 2.54)
Poor self-rated health	5.61	(3.17, 9.95)	2.46	(1.35, 4.51)
Low vitality	2.88	(1.79, 4.61)	1.05	(0.63, 1.76)

### 3.2 Contribution of prognostic indicators to persistent problems

The proportion of persistent problems at 12 months associated with each factor, calculated using PAFs, is shown in Table 3. All proportions fell after adjustment, but many of the adjusted figures were high: five prognostic indicators had statistically significant proportions, and six were above 40%. The highest proportion was for high pain intensity, indicating that in 68% of LBP patients with a poor outcome, outcome is related to high baseline pain intensity, regardless of the presence of the other risk factors. The next highest proportion was for not being in employment (64%). Poor self-rated health, and high functional disability, upper body pain and pain bothersomeness all also had proportions over 40% (although non-significant for upper body pain and bothersomeness).

**Table 3:** Adjusted population attributable fractions (PAFs) for the association between baseline risk factors and outcome at 12-months (*n* = 389).

Risk factor	Unadjusted	Adjusted	
	PAF (%)	PAF (%)	(95% CI)
Gender (F)	–	–	–
Age group (older)	–	–	–
Less education	34.7	11.6	(−28.1, 43.1)
Not being in employment	75.1	63.8	(32.6, 81.9)
Dissatisfaction with work status	54.9	32.2	(−5.8, 59.5)
Work absence	53.5	38.0	(11.8, 59.3)
Previous history	–	–	–
Long duration	37.1	25.4	(−3.3, 49.8)
High functional disability	62.6	47.6	(13.5, 70.2)
High pain intensity	81.0	68.1	(27.1, 87.0)
Leg pain	57.0	21.5	(−47.9, 61.2)
Distal leg pain	24.5	− 0.4	(−33.0, 29.4)
Upper body pain	67.2	49.2	(−17.2, 79.5)
Bothersomeness	72.6	45.8	(−16.5, 76.3)
Anxiety	55.4	32.5	(−2.2, 58.4)
Depression	42.3	19.5	(−8.8, 44.5)
Fear-avoidance	20.8	3.0	(−17.2, 25.2)
Catastrophising	52.0	20.6	(−15.5, 48.8)
Poor self-rated health	64.3	46.5	(12.4, 69.5)
Low vitality	34.0	2.7	(−27.6, 30.8)

Combining risk factors within domains showed that symptom severity had the highest cumulative effect (Table 4); people with both high pain and high functional disability comprised 72% of everyone with a poor outcome and were almost seven times more likely (RR 6.9) to have a poor outcome than people with neither high pain nor high disability. The cumulative proportion was 74% for the symptom severity domain, indicating that in almost three quarters of people with a poor outcome, that outcome is related to baseline symptom severity. Widespreadness of pain had a cumulative proportion of 70%. Pain affect had a lower cumulative proportion of 40% with pain cognition having a small effect (13%) on outcome.

**Table 4:** Cumulative effects of risk factors on poor outcome by domain.^a^

Baseline risk factor	CPG grades I–III at 12 months (%)	CPG grade IV^b^ at 12 months (%)	Adjusted RR	Adjusted PAF (%)
*Symptom severity*				
Neither high pain nor functional disability	57	9	1.00	
High pain only	22	17	3.10	11.8
High functional disability only	6	1	1.43	0.4
High pain and functional disability	15	72	6.90	62.0

Total				74.2

*Widespread pain*				
Neither leg nor upper body pain	13	1	1.00	
Leg pain only	17	7	1.91	3.5
Upper body pain only	25	13	3.06	8.8
Leg and upper body pain	45	78	3.75	57.4

Total				69.6

*Pain affect*				
Neither anxious nor depressed	67	20	1.00	
Anxious only	16	23	1.80	10.3
Depressed only	6	9	1.47	2.8
Anxious and depressed	12	48	2.34	27.4

Total				40.4

*Pain cognitions*				
Not catastrophising nor fear-avoidance	69	32	1.00	
Catastrophising only	11	29	1.24	5.7
Fear-avoidance only	11	3	0.46	−3.4
Catastrophising and fear-avoidance	8	36	1.40	10.4

Total				12.7

a For domains with more than two risk factors.

b Defined as poor outcome.

Combining the two individual risk factors with the largest proportions (pain and unemployment) resulted in a cumulative proportion of 85% (Table 5). 78% of the 69 patients with poor outcome had both high pain and unemployment at baseline compared to 11% of those with better outcomes.

**Table 5:** Cumulative effects of high pain intensity and not being in employment on poor outcome.

Baseline risk factor	CPG grades I–III at 12 months (%)	CPG grade IV^a^ at 12 months (%)	Adjusted RR	Adjusted PAF (%)
Neither high pain nor unemployed	48	4	1.00	
Unemployed only	15	6	3.52	4.2
High pain only	26	12	3.31	8.1
High pain and unemployed	11	78	14.41	72.8

Total				85.1

a Defined as poor outcome.

## 4 Discussion and conclusions

We have demonstrated that a range of factors significantly increase the risk of a poor outcome in patients visiting their GP with LBP. These large risks, in combination with high risk factor prevalence in this population, leads to substantial proportions of outcome related to the factors, even after adjustment. Potentially treatable factors such as high back pain intensity and concurrent pain in the upper body (multiple site pain) made large contributions to prognosis (i.e. a large proportion of the poor outcome was related to these factors), and this is consistent with the pain intensity being an important target for primary care intervention. High pain at baseline and not being in employment together were key factors predicting poor outcome. This highlights that LBP is not just a problem in people currently employed. Combining risk factors from within domains showed that risk factors rarely occur in isolation in these patients, and where predicting prognosis is the aim, little may be added by measuring a range of factors with substantial overlap, such as functional disability and pain, or leg pain and upper body pain.

All the individual prognostic indicators highlighted as statistically significant and independent in this analysis have previously been found to be important. Examples of these previous studies are: unemployment (Reis et al., 1999), work absence (Schiøttz-Christensen et al., 1999), episode duration (Burton et al., 2004, van den Hoogen et al., 1998, Mallen et al., 2007), functional disability (Carey et al., 2000, Coste et al., 1994, van den Hoogen et al., 1998), pain intensity (Croft et al., 1998, Mallen et al., 2007), anxiety (Lanier and Stockton, 1988, Mallen et al., 2007), and self-rated health (Deyo and Diehl, 1988). This overall consistency with other research is evidence towards the generalisability of the findings. Factors not highlighted as important in this study included fear- avoidance and catastrophising. The brief measurement method used could have impacted on the findings, but recent reviews (Pincus et al., 2006, Mallen et al., 2007), and a study of similar primary care back pain consulters (Foster et al., 2010), have not clearly identified fear-avoidance beliefs or catastrophising as being indicators of outcome in primary care, although other work suggests that these factors are important in the pain experience (Thibault et al., 2008).

Some factors previously identified as prognostic indicators became non-significant following adjustment, such as depression and upper body pain (indicating multiple pain sites); this is not necessarily a contradiction to previous research, as many studies have not adjusted for potential confounders. (Mallen et al., 2007) Furthermore, we have studied patients who consulted about LBP but who often had pain in other sites; we have focused on the outcome of their LBP, but predictors of pain prognosis generally may be different among all primary care consulters with multiple pains. Previous history of back pain has been highlighted as a prognostic indicator in other studies (Mallen et al., 2007), but this was not supported here, probably due to the very high proportion of the sample with prior back pain (87%). Although a wide range of prognostic indicators were included here, other factors have been identified elsewhere (e.g. Mallen et al., 2007, Foster et al., 2010) and it would be useful to examine these. Replication in other samples, perhaps with recent onset back pain, would be useful, as the current sample was mixed, and contained many people with long duration of pain.

A strength of this study is presentation of the contribution of prognostic factors to poor outcome through the use of adjusted PAF calculations. Whilst adjustment for confounding is considered essential for models of outcome prediction, adjustment of PAFs is rare. Table 3 demonstrates that proportions can change substantially following adjustment, and presentation of unadjusted proportions would considerably overestimate the contribution of several factors. Although there was loss to follow-up in the study, the sample is representative of baseline responders. Attrition biases are unlikely to substantially influence the RRs reported here, as comparisons are within the sample. However, as the proportions corresponding to each factor are based on associations and risk factor prevalence, these may be affected. In this analysis, 47% of the sample had high pain intensity at baseline, compared to 46% in the total baseline sample; loss to follow-up is therefore unlikely to have affected the proportions reported. However, initial response to the survey was 65%, and it is likely that non-responders were different to baseline responders. The impact of this is difficult to assess due to lack of information, but it is likely that the prevalence of prognostic indicators would be lower among non-responders. However, even a 10% change in the prevalence of the prognostic indicator would only make a difference in the proportion of poor outcome associated with pain intensity of around 4% (e.g. reducing high pain intensity prevalence from 47% to 37% would lead to an unadjusted proportion of 77% compared with 81% in Table 3), indicating that our results are likely to be broadly generalisable. Comparisons are also difficult to make with other samples due to the different measures used, lack of information about prevalence of prognostic indicators, and the inability to produce adjusted figures without the original data. As proportions differ according to the prevalence of exposure and strength of association, estimates of the potential contributions of prognostic indicators should be made for individual settings.

The factors included in the models presented here either themselves provide targets for intervention, which could substantially shift population outcomes, or identify the sub-groups where other interventions could achieve such changes. The factor with the largest contribution in this paper – high pain intensity – is theoretically modifiable in primary care, e.g. using analgesic medication or spinal manipulation (Chou et al., 2007). Although such treatments rarely provide complete pain relief, as the risk factor is common (47% of this sample), even slight improvements in pain management leading to a small shift in mean pain levels could have an important influence on the LBP population. Targeting pain may seem obvious, but the fact that many patients still experience pain after primary care management (Hestbaek et al., 2003) indicates room for improvement. Targeting such a common factor may also conflict with the expectation that we should be looking for less common factors to identify the minority who are at risk for long-term problems, but our whole population approach (in this case a primary care population) indicates that the most benefit for the population would be reached by targeting a group of people with a common factor such as pain. This finding should be considered alongside suggestions that a dominant focus on pain as a target for “cure” might mean that back pain is being overtreated (Deyo et al., 2009). However, the ‘overtreatment’ referred to is predominantly epidural steroid injections, opioids and lumbar magnetic resonance imaging, none of which are first line management approaches in primary care populations (Van Tulder et al., 2006, Airaksinen et al., 2006). Other interventions may be warranted which are less focused on the pain itself, and which may also reduce pain levels, such as activity-based interventions, work rehabilitation or cognitive behavioural approaches.

The factor identified with the next highest contribution – not being in employment – is more problematic within this setting. In occupational settings, enabling return to work in back pain sufferers is commonly addressed (Nguyen and Randolph, 2007), and our findings justify that priority. However, people without current employment would not be addressed in an occupational setting. In current UK primary care, GPs rarely have any influence over return to work (if employed) or return to employment (if unemployed). Our findings justify the UK government initiative addressing health, work and wellbeing (http://www.workingforhealth.gov.uk/). A multifactorial approach, acknowledging social influences on LBP, would likely also be beneficial in other settings where health care and employment are separated.

The PAF calculations are important intervention strategies for LBP in primary care as a whole, as they estimate the relative contribution of various factors to outcome. Studies in LBP usually only present measures of association (RRs, ORs), but these vary in overall contribution according to how common the risk factors are. Using PAF type calculations provides a useful way of comparing risk factors of differing prevalence at a population level. While this does not necessarily address the problems of the small proportion of primary care consulters with specific back problems (Deyo and Phillips, 1996), it may enable healthcare providers to identify useful areas or sub-groups for intervention which could shift outcomes overall within a primary care population. Given that LBP patients represent a significant proportion of all sufferers in primary care, this is therefore a sensible arena for public health secondary prevention of persistent LBP, and figures such as those presented in this paper can facilitate prioritisation of scarce health resources.
